# Raloxifene adjunctive therapy for postmenopausal women suffering from chronic schizophrenia: a randomized double-blind and placebo controlled trial

**DOI:** 10.1186/2008-2231-22-55

**Published:** 2014-07-10

**Authors:** Gilda Kianimehr, Farzad Fatehi, Sara Hashempoor, Mohammad-Reza Khodaei-Ardakani, Farzin Rezaei, Ali Nazari, Ladan Kashani, Shahin Akhondzadeh

**Affiliations:** 1 Psychiatric Research Center, Roozbeh Hospital, Tehran University of Medical Sciences, Tehran 13337, Iran; 2 Shariati Hospital, Neurology Department, Tehran University of medical Sciences, Tehran, Iran; 3 Razi Psychiatric Hospital, University of Social Welfare and Rehabilitation, Tehran, Iran; 4 Qods Hospital, Kurdistan University of Medical Sciences, Sanandaj, Iran; 5 Infertility Ward, Arash Hospital, Tehran University of Medical Sciences, Tehran, Iran; 6 Psychiatric Research Center, Roozbeh Psychiatric Hospital, Tehran University of Medical Sciences, South Kargar Street 13337, Tehran, Iran

**Keywords:** Schizophrenia, Menopause, Raloxifene, Selective estrogen receptor modulators

## Abstract

**Background:**

Cumulative evidence from epidemiological, preclinical and clinical studies suggests estrogens may have psychoprotective effects in schizophrenic patients. Selective Estrogen Receptor Modulators could have therapeutic benefits in schizophrenia for both sexes without being hazardous to gynecological tissues or having feminizing effects. Few studies have been conducted regarding the effects of raloxifene on postmenopausal women suffering from schizophrenia. We conducted this placebo-controlled trial to compare the add-on effect of raloxifene to risperidone versus risperidone with placebo.

**Methods:**

This was an 8-week, parallel-group, placebo-controlled trial undertaken at two universities affiliated psychiatric Hospitals in Iran. Forty-six postmenopausal women with the definite diagnosis of schizophrenia were enrolled in the study. Patients received risperidone (6 mg/day in 3 divided doses) combined with either placebo (N = 23) or 120 mg/day of raloxifene (N = 23) for 8 weeks. Patients were assessed by a psychiatrist at baseline and at 2 and 8 weeks after the start of medical therapy. Efficacy was defined as the change from baseline to endpoint in score on Positive and Negative Syndrome Scale (PANSS).

**Results:**

For PANSS scores, the main effect comparing two types of intervention was not significant [F (1, 48) = 1.77, p = 0.18]. For positive subscale scores, there was marginal significant interaction between intervention type and time [F (2, 47) = 2.93, p = 0.06] and there was substantial main effect for time [F (2, 47) = 24.39, p = 0.001] within both groups showing reduction in positive subscale scores across the three time periods. In addition, the main effect comparing two types of intervention was significant [F (1, 48) = 3.78, p = 0.02]. On the other hand, for negative subscale scores, the main effect comparing two types of intervention was not significant [F (1, 48) = 1.43, p = 0.23]. For general subscale scores, the main effect comparing two types of intervention was not significant [F (1, 48) = 0.03, p = 0.86].

**Conclusions:**

According to our findings, raloxifene as an adjunctive treatment to risperidone was only superior in improvement of positive symptoms and it was not effective in treating negative and general psychopathology symptoms.

**Trial registration:**

The trial was registered at the Iranian registry of clinical trials: IRCT201205131556N42

## Background

Cumulative evidence from epidemiological, preclinical and clinical studies suggests estrogens may provoke psychoprotective effects in schizophrenic patients [1]. In addition, Selective Estrogen Receptor Modulators (SERMs) could have therapeutic benefits on both sexes with schizophrenia without any hazard to gynecological tissues or feminizing effects [1]. There are several lines of evidence that support the estrogen hypothesis of schizophrenia [2]. Most antipsychotics cause prolactin-inducing effects, and ensuing negative feedback on estrogen levels [2]. Sex differences in the incidence, onset and course of schizophrenia have led to the hypothesis that estrogens play a protective role in the pathophysiology of this disorder [3]. Estrogen treatment may boost the recovery of schizophrenia in women. However, adverse effects on uterine and breast tissue and other physical side effects may limit the long-term therapeutic use of estrogen [4-6]. Raloxifene hydrochloride is a selective estrogen receptor modulator that acts as an estrogen antagonist in breast tissue and may have agonistic actions in the brain, potentially offering mental health benefits with few estrogenic side effects [4].

Prognosis and severity of schizophrenia is different between males and females and as a result, it is suggested that estrogen may play a role in the pathogenesis of schizophrenia [1,3]. sex differences is a fascinating aspect of schizophrenia, which have been described for nearly all features of the illness, including the peak age of onset, symptoms and treatment response [7]. Estradiol and some SERMs are neuroprotective in a variety of experimental models of neurodegeneration; they could lessen the inflammatory response of glial cells, decrease anxiety and depression, and endorse cognition. Moreover, they are able to modulate synaptic plasticity in the hippocampus of rodents [8]; however, long term estrogen application may cause significant complications especially in menopausal women. Therefore, administration of Selective Estrogen Receptor Modulators (SERMs) may reduce such side effects. Raloxifene is a SERM that has similar dopaminergic and serotonergic effects as estrogen. Few studies have been conducted regarding the effects of raloxifene on postmenopausal women suffering from schizophrenia [9,10]. However, the sample size of these studies was relatively small [9.10]. We conducted this placebo-controlled trial to compare the add-on effect of raloxifene to risperidone in comparison with risperidone and placebo.

## Methods

### Trial organization

This was an 8-week, parallel-group, placebo-controlled trial undertaken at two universities affiliated psychiatric Hospitals in Iran (Roozbeh and Razi Hospital) from June 2012 to December 2013. The trial protocol was approved by the Institutional Review Board (IRB) of Tehran University of Medical Science (Grant No: 17215) and was conducted in agreement with the Declaration of Helsinki and its subsequent revisions. The trial was registered at the Iranian registry of clinical trials (http://www.irct.ir; registration number: (IRCT201205131556N42). Written informed consent was obtained from all eligible patients and/or their legally authorized representatives. Patients were informed that they are free to withdraw from the study at any time without any influence on their relationship with their health care provider.

### Study population

Forty-six postmenopausal women with the definite diagnosis of schizophrenia were enrolled in the study. The diagnosis of schizophrenia was made according to DSM-IV TR. Diagnosis was made based on a Structured Clinical Interview for DSM-IV-TR Axis I Disorders (SCID) and was confirmed with chart review and senior physician interview. Inclusion criteria were postmenopausal (be in postmenopause is defined as period of one year spontaneous amenorrhea, a serum FSH level of >20 and clinical symptoms such as hot flashes,…) women who met the diagnostic criteria for schizophrenia according to DSM IV-TR consisting of Positive and Negative Syndrome Scale (PANSS) [11] score above 60 and disease onset over 2 years. Exclusion criteria were the concurrent presence of neurologic or organic disorders; IQ lower than 70; illicit drug use in past 6 months, consumption of antipsychotic drugs in the previous week or a long-acting antipsychotic drug in previous 2 months, receiving any antidepressant and mood stabilizer drugs during the trial, former hormone replace therapy, electroconvulsive therapy in past 2 weeks; endocrine abnormality; hepatic or renal dysfunction; and history of thromboemboli, abnormal uterine bleeding or breast and uterine cancer, and stroke.

### Study design

Forty-six postmenopausal women with DSM-IV-TR diagnosed chronic schizophrenia received risperidone (6 mg/day in 3 divided doses) (Janssen Pharmaceuticals, Toronto, Canada; 2 mg tablet) combined with either placebo (N = 23) or 120 mg/day of raloxifene (Eli Lilly) (N = 23) for 8 weeks. Patients were assessed by a psychiatrist at baseline and at 2 and 8 weeks after the start of medical therapy. Efficacy was defined as the change from baseline to endpoint in score on PANSS.

### Outcome measurement

PANSS is one of the most important rating tools for computing symptom severity in patients with schizophrenia. It comprises 3 subscales (positive scale, negative scale, and general psychotherapy scale) with 30 items in total ranging in score between 1 and 7 which leads to a minimum total score of 30. PANSS has been used in a number of trials in Iran [12-15]. The mean decline in PANSS scale as well as subscales from baseline was regarded as the main outcome measure of response in schizophrenia to treatment. The scale was measured at baseline, week 2 and week 8. Efficacy will be defined as change from baseline to endpoint in score on PANSS. Adverse events were evaluated using a 25 –item checklist and Extra-pyramidal Symptoms Rating Scale (ESRS) [16].

### Statistical analysis

SPSS software, version 20.0 for Windows, was used to analyze the data. Continuous data are presented as mean ± standard deviation (SD). We used Kolmogorov-Smirnov Z test to determine the normal distribution of all continuous variables and due to normal distribution of PNSS score and subscales (positive, negative, general), we used parametrical tests to analyze data. A mixed between-within subjects analysis of variance was conducted to assess the impact of two different interventions (Risperidone + Raloxifene, Risperidone + placebo) on patient scores on PANSS score, positive subscale, negative subscale, and general psychotherapy subscale, across three time periods (the start of study, week 2, and week 8).

## Results

**Figure 1:**
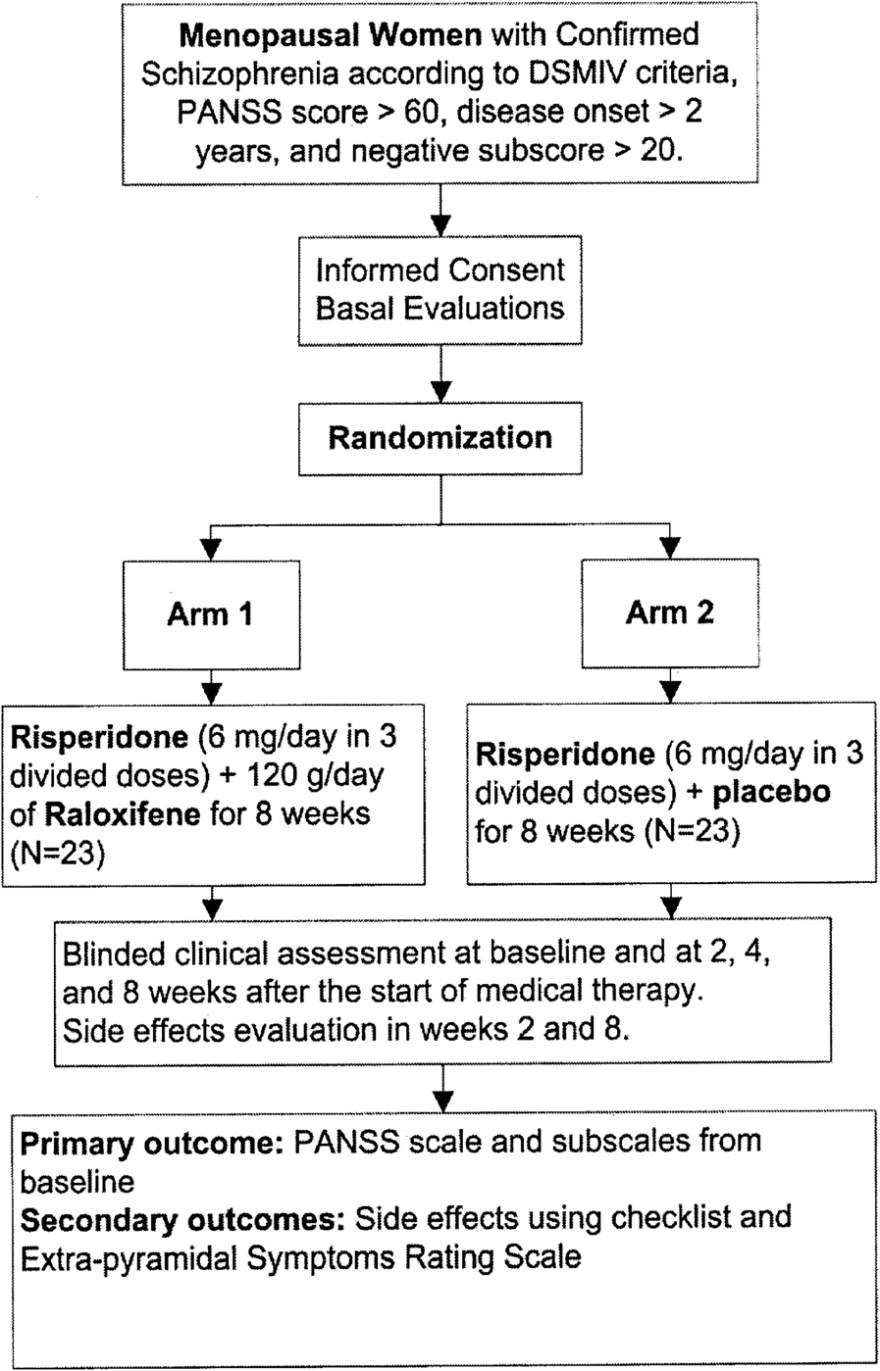
Trial flow diagram of adjunctive Raloxifene versus placebo.

Figure 1 shows flow diagram of the patients. The mean age (±SD) of the raloxifene group was 61.96 ± 4.49 years versus 60.44 ± 5.28 years in the placebo group (non significant). Demographic data of the patients is presented in Table 1.

**Table 1:** Demographic data of the raloxifene group versus the placebo group

		**Risperidone + Raloxifene group (N = 25)**	**Risperidone + Placebo group (N = 25)**	**p-value**
Age (±SD)		61.96 ± 4.49	60.44 ± 5.28	p > 0.05
Education	Illiterate	4 (36.36%)	7 (63.64%)	p > 0.05
	School	17 (51.52%)	16 (48.48%)	
	University	3 (50%)	3 (50%)	
Positive family history		13 (42%)	15 (60%)	p > 0.05
Age of onset		34.96 ± 11.69	29.40 ± 8.57	p > 0.05
Duration		17.24 ± 12.03	13.64 ± 12.41	p > 0.05
Previous treatment	Risperidone	7 (41.12%)	10 (58.88%)	p > 0.05
	Other atypical	3 (75.0%)	1 (25.0%)	
	Typical	11 (50.0%)	11 (50.0%)	
	Combination	4 (57.14%)	3 (42.86%)	
IQ	Normal	24 (52.17%)	22 (47.83%)	p > 0.05
	Borderline	1 (25.0%)	3 (75.0%)	

**Figure 2:**
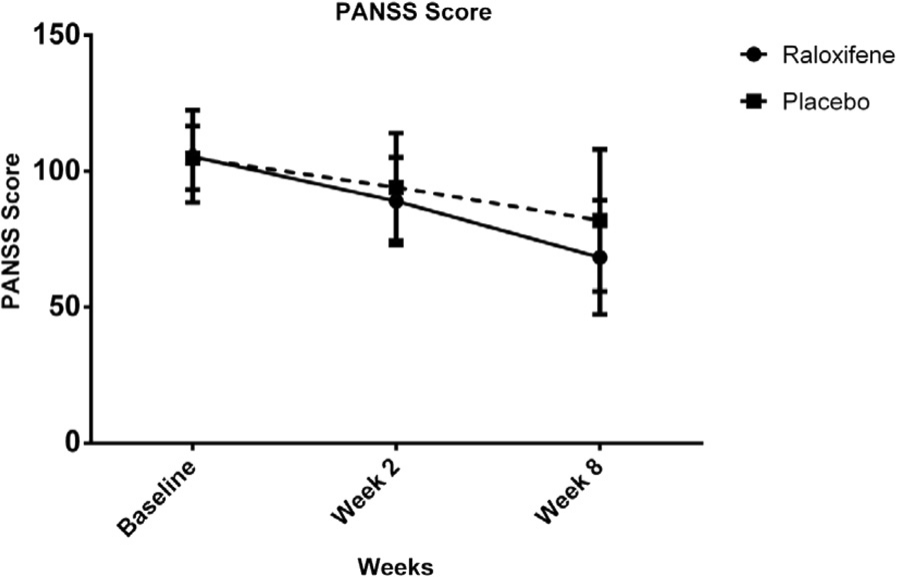
PANSS scores for the intervention type across three time periods (Raloxifene group: solid line, Placebo group: dashed line).

For PANSS scores, there was no significant interaction between intervention type and time [Wilks’ Lambda = 0.89, F (2, 47) = 2.79, p = 0.07]. There was substantial main effect for time [Wilks’ Lambda = 0.36, F (2, 47) = 42.56, p < 0.01] within both groups showing a reduction in PANSS scores across three time periods (Table 2, Figure 2). Furthermore, the main effect comparing two types of intervention was not significant [F (1, 48) = 1.77, p = 0.18].

**Table 2:** PANSS and subscale scores for the intervention type across three time periods

		**Risperidone + Raloxifene (n = 25)**	**Risperidone + Placebo (n = 25)**
	**Time period**	**Mean ± SD**	**Mean ± SD**
PANSS score	Week 0	105.52 ± 16.96	105.00 ± 11.68
	Week 2	89.08 ± 16.14	94.16 ± 19.78
	Week 8	68.32 ± 21.04	82.00 ± 26.14
Positive subscale	Week 0	25.76 ± 8.00	26.68 ± 7.04
	Week 2	20.92 ± 5.41	24.28 ± 6.33
	Week 8	14.32 ± 4.33	21.12 ± 7.07
Negative subscale	Week 0	28.04 ± 8.64	30.76 ± 9.09
	Week 2	24.88 ± 8.76	28.12 ± 10.88
	Week 8	19.60 ± 8.66	23.08 ± 12.08
General Psychopathology subscale	Week 0	50.44 ± 9.92	47.52 ± 6.46
	Week 2	43.56 ± 9.30	41.88 ± 9.76
	Week 8	34.56 ± 10.78	37.84 ± 11.89

**Figure 3:**
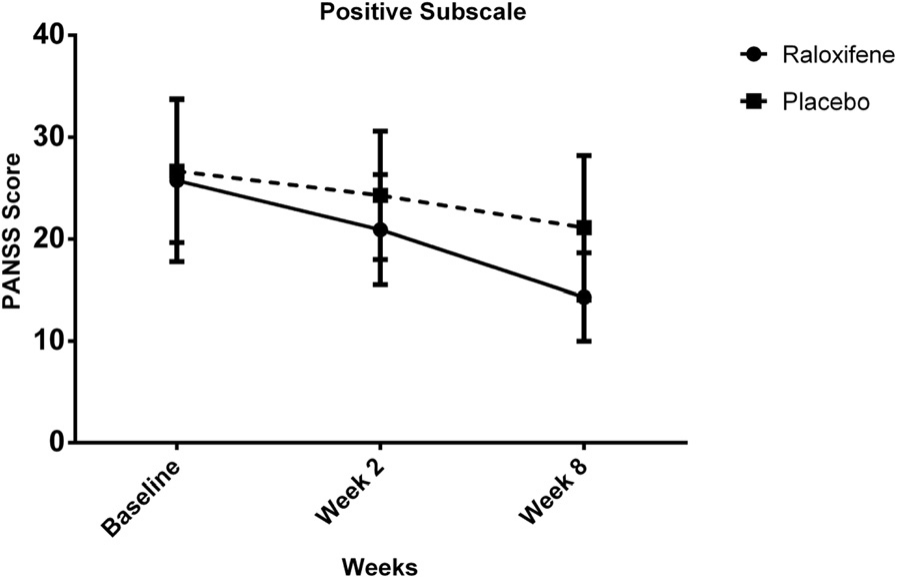
Positive subscale scores for the intervention type across three time periods (Raloxifene group: solid line, Placebo group: dashed line).

For positive subscale scores, there was marginal significant interaction between intervention type and time [Wilks’ Lambda = 0.89, F (2, 47) = 2.93, p = 0.06] and there was substantial main effect for time [Wilks’ Lambda = 0.44, F (2, 47) = 24.39, p < 0.01] within both groups showing a reduction in positive subscale scores across the three time periods (Table 2, Figure 3). In addition, the main effect comparing two types of intervention was significant [F (1, 48) = 3.78, p = 0.02] suggesting superior effectiveness of Risperidone plus Raloxifene over Risperidone plus Placebo.

**Figure 4:**
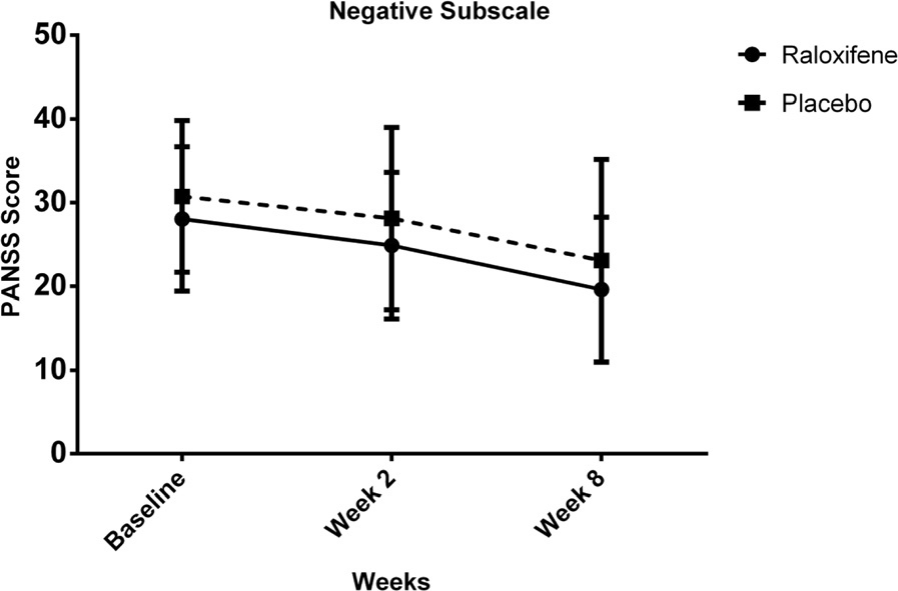
Negative subscale scores for the intervention type across three time periods (Raloxifene group: solid line, Placebo group: dashed line).

For negative subscale scores, there was no significant interaction between intervention type and time [Wilks’ Lambda = 0.99, F (2, 47) = 0.12, p = 0.73]. There was substantial main effect for time [Wilks’ Lambda = 0.46, F (2, 47) = 55.65, p < 0.01] within both groups showing a reduction in negative subscale scores across three time periods (Table 2, Figure 4). Additionally, the main effect comparing two types of intervention was not significant [F (1, 48) = 1.43, p = 0.23] suggesting no difference in effectiveness of the two therapies on negative subscale scores.

**Figure 5:**
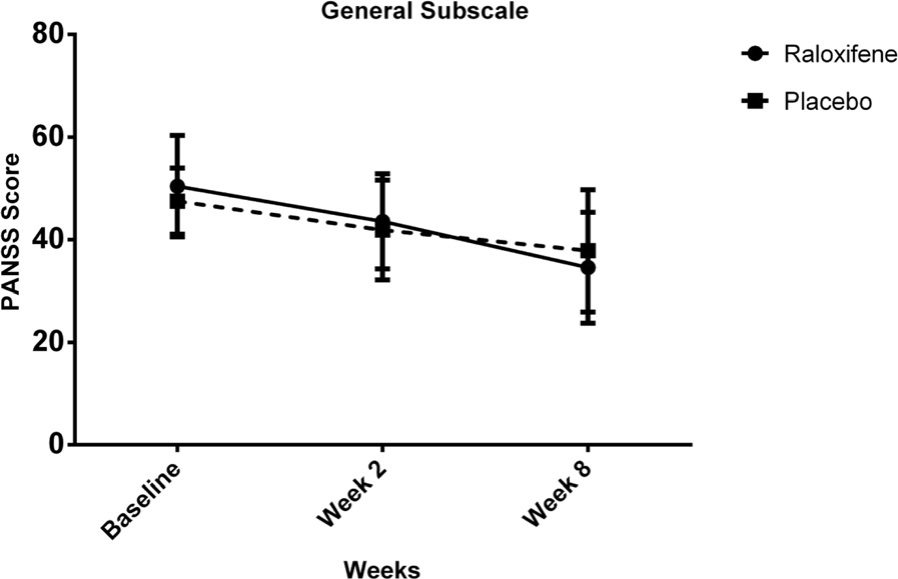
General Psychopathology subscale scores for the intervention type across three time periods (Raloxifene group: solid line, Placebo group: dashed line).

For general subscale scores, there was significant interaction between intervention type and time [Wilks’ Lambda = 0.78, F (2, 47) = 6.78, p < 0.01]. There was substantial main effect for time [Wilks’ Lambda = 0.34, F (2, 47) = 46.13, p < 0.01] within both groups showing a reduction in general subscale scores across the three time periods (Table 2, Figure 5). However, the main effect comparing two types of intervention was not significant [F (1, 48) = 0.03, p = 0.86] suggesting no difference in the effectiveness of the two therapies on general subscale scores.

Main adverse events included constipation (3 patients in Raloxifene group and 2 patients in placebo group), tremor (1 in each group), dry mouth (2 patients in raloxifene group), restless leg syndrome (1 patient in Raloxifene group), drowsiness (2 patients in placebo group), and loss of appetite (1 patient in placebo group). In addition, there was no ESRS difference between the two groups at weeks 2 and 8 [F (1, 48) = 1.04, p = 0.30].

## Discussion

Adjunctive therapy and polypharmacy is used routinely for several chronic diseases. Medications with different mechanisms of action are used in polypharmacy. The use of adjunctive therapy in schizophrenia has been a major concern especially in recent years. The potential therapeutic efficacy of estrogens in schizophrenia is being identified and Selective Estrogen Receptor Modulators (SERMs) seem to be a better option in view of safety concerns. According to our findings, Raloxifene as an adjunctive treatment to risperidone was only superior for positive symptoms and it was not effective in treating negative and general psychopathology symptoms.

Recently, some studies have pointed to the adjunctive efficacy of raloxifene in improving schizophrenic symptoms [17]. In a 12-week, double-blind, randomized, placebo-controlled study [10], 33 postmenopausal women with schizophrenia were randomized to receive either adjuvant raloxifene (16 women) or adjuvant placebo (17 women) for three months. The addition of raloxifene produced significant differences in some aspects of memory and executive function in patients treated with raloxifene. In another 12-week, double-blind, randomized, placebo-controlled study, 33 postmenopausal women with schizophrenia and prominent negative symptoms were randomized to either adjunctive raloxifene or adjunctive placebo for 12 weeks [9]. The addition of raloxifene (60 mg/d) to regular antipsychotic treatment significantly reduced negative, positive, and general psychopathology symptoms compared to the placebo group.

In a randomized clinical trial [4], 35 postmenopausal women with schizophrenia received 120 mg/day adjunctive raloxifene compared to either adjunctive raloxifene HCl (60 mg/day) or placebo. A significantly greater recovery of total and general PANSS symptoms was noted in patients receiving 120 mg/day adjunctive raloxifene compared to either 60 mg/day adjunctive raloxifene HCl or placebo. In addition, adjunctive Raloxifene has been successfully used in single case reports for menstruating women suffering from resistant schizophrenia. A 29-year-old woman, with drug-resistant schizophrenia, experienced significant improvement in socio-occupational functioning, with reduction in symptom severity, over a 7-month follow-up period, [18].

It is significant that we encountered no adverse effects in the raloxifene group compared to the placebo group. The safer raloxifene profile compared with estrogenic compounds, makes it a preferred drug when adjunctive hormonal therapy of schizophrenia for post-menopausal women is considered. The lack of adverse effects on breast and uterine tissue is an imperative advantage of Raloxifene over estrogen [19].

The ovarian hormone 17β-estradiol acts in the central nervous system to regulate neuroendocrine events and reproduction. Estradiol controls gene expression, neuronal survival, neuronal and glial differentiation, and synaptic transmission and has anti-inflammatory, protective and reparative properties in the brain [20,21]. A number of studies seem to indicate that raloxifene acts on brain dopamine and serotonin systems in a way that is similar to that of conjugated estrogens [22].

It is proposed that conjugated estrogens may exercise their therapeutic potential either by modulating brain neurotransmission or through neuroprotective effects. On the other hand, the incidence of schizophrenia in men is consistently observed to be approximately 1.5 times higher than its in women [23] and there is a male predominance in incidence in the early twenties, but a female predominance in older middle age [24]. Estrogen may have an impact on schizophrenia by several mechanisms. Short-term, it seems to have fast membrane effects by modifying functional activity in the dopaminergic synapse; for longer periods, it may appear to have genomic effects by altering synthesis at dopamine receptors. There is also evidence to suggest that estrogen alters serotonergic systems. Estrogen can also promote neuronal regeneration and block mechanisms of neuronal death [4]. In addition, numerous studies have described association between serotonin 1A receptor and major psychiatric disorders, such as schizophrenia and bipolar disorder [25]. Both serotonin and estrogen have been involved in modulation of mood and cognition. Even though substantial functional relations between estrogen and serotonin are recognized, the nature of their relationship has not been fully illuminated [26]. This study has some limitations which should be considered. The sample size was relatively small and the study course and follow-up period were relatively short. We did not measure the effect of raloxifene on menopausal symptoms as well. Furthermore, lack of cognitive assessments is another limitation of the present study.

## Conclusions

In conclusion, it seems that according to our findings, raloxifene as an adjunctive treatment was shown to be superior to placebo in improving the positive symptoms in patients receiving risperidone. However, this drug did not show a benefit in treating the negative and general symptoms of schizophrenia when was added to risperidone therapy.

## Abbreviations

SERMs: Selective estrogen receptor modulators; PNASS: Positive and negative syndrome scale.

## Competing interests

No conflict of interest exists for any of the authors associated with the manuscript and there was no source of extra-institutional commercial funding. The funding organization had no role in the design and conduct of the study; in the collection, analysis, and interpretation of the data; or in the preparation, review, or approval of the manuscript and the decision to submit the paper for publication.

## Authors’ contributions

GK, SH and AN: Sample collection, FF: Statistical Analysis, Article writing, SA, LK, MK and FR: Designer and project manager, Article writing. All authors read and approved the final manuscript.
